# A walk in the PARC: developing and implementing 21st century chemical risk assessment in Europe

**DOI:** 10.1007/s00204-022-03435-7

**Published:** 2023-01-16

**Authors:** P. Marx-Stoelting, G. Rivière, M. Luijten, K. Aiello-Holden, N. Bandow, K. Baken, A. Cañas, A. Castano, S. Denys, C. Fillol, M. Herzler, I. Iavicoli, S. Karakitsios, J. Klanova, M. Kolossa-Gehring, A. Koutsodimou, J. Lobo Vicente, I. Lynch, S. Namorado, S. Norager, A. Pittman, S. Rotter, D. Sarigiannis, M. J. Silva, J. Theunis, T. Tralau, M. Uhl, J. van Klaveren, L. Wendt-Rasch, E. Westerholm, C. Rousselle, P. Sanders

**Affiliations:** 1 German Federal Institute for Risk Assessment (BfR), Max-Dohrn-Str. 8-10, 10589 Berlin, Germany; 2 French Agency for Food, Environmental and Occupational Health and Safety (ANSES), 94701 Maisons-Alfort, France; 3 National Institute for Health and Environment (RIVM), Bilthoven, The Netherlands; 4 German Environment Agency (UBA), Wörlitzer Platz 1, 06844 Dessau, Germany; 5 National Centre for Environmental Health, Instituto de Salud Carlos III (ISCIII), Madrid, Spain; 6 Santé Publique France (SpFrance), 12, Rue du Val D’Osne, 94415 St. Maurice, France; 7 Department of Public Health, University of Naples Federico II (UNINA), Naples, Italy; 8 Aristoteles University Thessaloniki (AUTH), Thessaloniki, Greece; 9 Masaryk Uinversity, Recetox, Kotlarska 2, 61137 Brno, Czechia; 10 General Chemical State Laboratory of Greece, Athens, Greece; 11 European Environment Agency, Kongens Nytorv 6, 1050 Copenhagen K, Denmark; 12 School of Geography, Earth and Environmental Sciences, University of Birmingham (UoB), Edgbaston, Birmingham, B15 2TT UK; 13 National Institute of Health Dr. Ricardo Jorge (INSA), Avenida Padre Cruz, 1649-016 Lisbon, Portugal; 14 European Commission, DG Research and Innovation, Orban 09/199, 1049 Brussels, Belgium; 15 VITO (Flemish Institute for Technological Research), Boeretang 200, 2400 Mol, Belgium; 16 Austrian Federal Environments Agency, Vienna, Austria; 17 Swedish Chemicals Agency (KemI), Vasagatan 12D, 172 67 Sundbyberg, Sweden

**Keywords:** Next-generation risk assessment (NGRA), Chemicals, Safety assessment, Exposure assessment, Hazard characterisation, Human biomonitoring (HBM), New approach methods (NAM)

## Abstract

Current approaches for the assessment of environmental and human health risks due to exposure to chemical substances have served their purpose reasonably well. Nevertheless, the systems in place for different uses of chemicals are faced with various challenges, ranging from a growing number of chemicals to changes in the types of chemicals and materials produced. This has triggered global awareness of the need for a paradigm shift, which in turn has led to the publication of new concepts for chemical risk assessment and explorations of how to translate these concepts into pragmatic approaches. As a result, next-generation risk assessment (NGRA) is generally seen as the way forward. However, incorporating new scientific insights and innovative approaches into hazard and exposure assessments in such a way that regulatory needs are adequately met has appeared to be challenging. The European Partnership for the Assessment of Risks from Chemicals (PARC) has been designed to address various challenges associated with innovating chemical risk assessment. Its overall goal is to consolidate and strengthen the European research and innovation capacity for chemical risk assessment to protect human health and the environment. With around 200 participating organisations from all over Europe, including three European agencies, and a total budget of over 400 million euro, PARC is one of the largest projects of its kind. It has a duration of seven years and is coordinated by ANSES, the French Agency for Food, Environmental and Occupational Health & Safety.

## Introduction

Risk assessment approaches underpinning regulatory decisions with regard to chemical substances have been set to protect both human and environmental health. These approaches have been serving their purpose reasonably well. However, the regulatory risk assessment systems currently in place are facing various challenges. These challenges are diverse, ranging from a growing number of chemicals that need to undergo risk assessment via chemical mixtures, aggregate exposure and complex health effects like endocrine disruption or developmental neurotoxicity to new materials such as advanced materials. In order to address these challenges in a scientifically sound manner, innovative approaches for chemical exposure, hazard and ultimately risk assessment are urgently needed.

Human, animal and plant populations are exposed to a large number of chemical substances and a plethora of potential combinations. Current testing methods accepted by regulators rely to a large extent on animal testing. For ethical as well as economic reasons in vivo tests are not applicable to assess all sorts of combinations. Therefore, better performing approaches have to be developed so that health risks linked to exposure to combinations of chemicals can be assessed more efficiently. This involves not only hazard assessment but also human biomonitoring (HBM), environmental monitoring and exposure modelling, to ensure that comprehensive information on exposure is obtained. PARC is based on a number of European projects including but not limited to the SEURAT initiative (Daston et al. 2015; Gocht et al. 2015), EuroMix (Rotter et al. 2018), EuToxRisk (Escher et al. 2019; Krebs et al. 2020; Mone et al. 2020) or the European Joint Programme HBM4EU (Ganzleben et al. 2017; Kolossa-Gehring et al. 2023) but also unique as the concept of partnerships has recently been elaborated under the EU Horizon Europe framework. Furthermore, chemical substances are regulated under different legislative frameworks in Europe, depending on the intended use. Hence, risks to humans and to the environment for the same chemical are often assessed separately, in different regulatory frameworks and when addressing human health, the assessment does frequently not consider all sources of exposure mainly due to the different frameworks.

Traditional toxicity test methods frequently rely on the use of experimental animals. This has a number of implications: the results obtained with experimental animals are not necessarily relevant to human health risk assessment. Furthermore, there are important ethical and economic considerations to move away from animal use for toxicity testing. Hence, chemical risk assessment is in high demand of a paradigm shift.

Over the years, new concepts for chemical risk assessment have been proposed (Council 2007; Embry et al. 2014; Krewski et al. 2010; Pastoor et al. 2014). Overall, from a scientific and technological perspective, these concepts for NGRA are considered feasible (see (Luijten et al. 2022) and references therein). However, incorporating scientific progress and innovative approaches into hazard and exposure assessments in a manner that is workable as well as feasible from a legislative perspective has proved to be challenging. That is where the concept of a partnership between risk assessors, authorities and the scientific community can make a difference and help to implement innovations in testing and assessment into regulation.

**Fig. 1:**
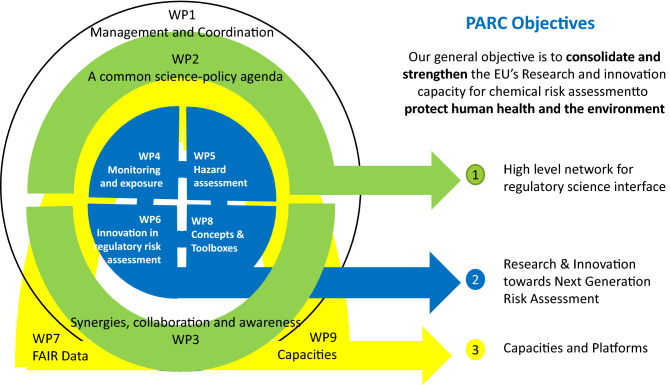
All WPs closely interact on transversal topics, and the R&I activities developed under WP4, WP5, WP6 and WP8 benefit from the support of WP2, WP3, WP7 and WP9

PARC has been developed to address the various challenges associated with innovating chemical risk assessment. It has an objective-oriented structure and, as illustrated in Fig. 1 is organised in nine work packages (WPs) working closely together to achieve three specific objectives (SOs):

SO1—EU and national risk assessors and regulatory entities come together with the scientific community in a cross-disciplinary network to set priorities for research and innovation (R&I) in chemical risk assessment;

SO2—European and national risk assessment entities and their scientific networks carry out a joint research and innovation programme to respond to the agreed priorities in chemical risk assessment;

SO3—European risk assessors, their scientific network and the wider stakeholder community have access to the research and innovation capacities required to implement innovative chemical risk assessment (see Fig. 1).

The PARC consortium comprises about 200 partners from twenty-eight countries, involving partners with relevant knowledge and expertise to meet the objectives of the consortium: from national agencies in charge of chemical risk assessment, national institutions of public/occupational health or public service and governmental organisations, to research institutes, universities, and hospitals or health care organisations and three EU Agencies (i.e. ECHA, EEA and ESFA). Together, they harness a broad range of expertise and disciplines required to achieve the goals set for PARC.

## High level network for regulatory science (SO1)

PARC, with its consortium involving both regulatory and academic scientists and being in close dialogue with EU and national regulatory agencies, is in a unique position to incorporate new advances in hazard and exposure assessment. In the wide range of activities foreseen in PARC, past (regulatory) experiences as well as scientific and technological advances required for transitioning towards NGRA are taken into account. In the context of PARC, NGRA refers to the concept of using data from New Approach Methodologies (NAMs) for chemical risk assessment. In its ideal the concept relies on tiered combinations of i*n silico* tools, complex in vitro systems, organ models and omics approaches in conjunction with physiologically based toxicokinetic modelling and complex exposure models. While the concept of NGRA comprises Adverse Outcome Pathways (AOPs) and quantitative AOPs as established tools for hazard assessment, it is not limited to these, nor does it exclude the use of in vivo data or histopathology. However, it puts a strong emphasis on using state-of-the-art systems and as such is predominantly mechanism-driven, and not driven by apical (toxicological) endpoints.

Classical approaches as well as NGRA rely on exposure data or models for their actual risk assessments. Successful regulatory examples of NGRA-elements already applied comprise assessments for cosmetic ingredients, selected cases of read-across and ED-assessments as well as NAM-based screenings under ToxCast, the Horizon 2020 project EUToxRisk or screenings of potential drug candidates in pharmaceutical industry. By aiming for putting NGRA into practice, PARC is addressing challenges related to the implementation of NAMs for chemical risk assessment, thereby overcoming challenges associated with substance-by-substance risk assessment, and substantially reducing negative impacts on biodiversity.

Achieving the global Sustainable Development Goals (https://sdgs.un.org/goals) also requires the development of sustainable chemistry, which should be safe for humans and the environment. In this context, PARC is establishing an EU-wide R&I hub of excellence composed of risk assessment and risk management bodies to support chemical risk assessment and risk management authorities at the national and European levels. This is instrumental to address current, emerging and novel chemical safety challenges and enabling the transition towards NGRA, in line with the European Green Deal’s zero-pollution ambition and in particular with the ‘Chemicals Strategy for Sustainability Towards a Toxic-Free Environment’ (EC 2020).

## Research and innovation towards NGRA (SO2; SO3)

PARC aims to strengthen the scientific basis of NGRA approaches to drive true innovation in chemical risk assessment. This is accomplished by reviewing current practices, and by developing and fostering transdisciplinary research that supports innovation related to both human health and the environment. PARC develops a holistic approach by developing tools and methods that will enable the integration of all main sources and routes of chemical exposure.

PARC will provide new tools and approaches that may be applied before registering or authorising new chemicals, to model actual exposure and risks, based on exposure scenarios and NAMs to identify potential toxic effects. PARC will foster 3R (Reduction, Refinement and Replacement) strategies and novel methods in toxicity testing such as in silico and in vitro models of relevance to humans. PARC will also provide innovative methods and capacities to monitor chemicals in appropriate human and environmental matrices. Analytical developments will be performed to identify non-targeted (NTS) and suspect screening methods to detect emerging contaminants and support the monitoring of real-world mixtures. By developing modelling approaches able to combine multiple sources and scenarios of exposure through different routes (oral, dermal, inhalation), PARC will contribute to the “one substance, one assessment” approach. Challenges related to the “one substance, one assessment” approach will be explored through reviews of risk assessment methodologies and regulatory experience, both within and across frameworks.

Additionally, PARC will contribute to facilitate the uptake and use of NGRA approaches in regulatory processes. In the different domains of PARC, methods and approaches will be applied in case studies, proficiency tests will be supported, and collaboration and interaction will be pro-actively sought with European and international bodies (*e.g.* EC-Joint Research Centre (JRC), Organisation for Economic Co-operation and Development (OECD) and World Health Organization (WHO)). This will allow for the characterisation of the NGRA approaches in terms of regulatory relevance, reliability and domain of application and move them forward on the path of standardisation.

PARC will take advantage of new tools and technologies to increase the efficiency of toxicity testing such as high-throughput in vitro test systems, omics, high content analysis and methods in computational toxicology. The development of adverse outcome pathways (AOP) (Ankley and Edwards 2018; OECD 2018b), which provide information on the causal links between a molecular initiating event (MIE), intermediary key events (KEs) and an adverse outcome (AO) of regulatory concern, offers the biological context to facilitate development of integrated approaches to testing and assessment (IATA) for regulatory decision-making (OECD 2020).

## WP2: a common science-policy agenda

One of the overall goals of PARC is to establish a sustainable cross-disciplinary network to set priorities for R&I in relation to chemical risk assessment. Over the years, various research initiatives in- and outside Europe have delivered innovative approaches for chemical risk assessment; however, their regulatory uptake has been limited, mainly due to a mismatch with the regulatory needs and the timely input of scientific results. The overall goals of this WP are to ensure the adequate links between research activities and regulatory needs through a prioritisation process, the establishment and maintenance of a central knowledge management platform, named ‘PARC*opedia*’, and a long- term strategic roadmap that safeguards the sustainability of PARC results and outputs after 7 years, entitled ‘PARC*route*’. It will also ensure high-level discussions at national and EU-level towards the sustainability of successful PARC activities after the 7 years.

### Task 2.1: priority setting

Targeted surveys, focused workshops and/or expert groups will be organised to gather information needs from policy-makers at both the EU and national level, as well as from stakeholders and experts involved in regulatory risk assessment activities. Together with partners involved in the various WPs of PARC, WP2 will ensure proper linkage between the high-level priorities of policy-makers at the EU and national level and the activities that will be implemented in PARC in response to these policy and regulatory needs. WP2 will also set up a common agenda at the science-policy interface, making PARC knowledge available and actively promoting its regulatory consideration, also working towards the sustainability of the network. Guided by policy and/or regulatory needs, WP2 will develop and implement a well-structured and transparent strategy aimed at the prioritisation of projects, substances and methodologies. Besides this prioritisation process, a ‘Rapid Response Mechanism’, based on HBM4EU’s experience will be set up (HBM4EU—European Joint Programme on Human Biomonitoring; https://www.hbm4eu.eu). This Rapid Response Mechanism is aimed at enabling national and European policy-makers to submit requests to the PARC Consortium for specific information on (combinations of) chemicals, thus ensuring that PARC responds to new and urgent needs for information in the EU policy community and at national level, outside of the formal timeframes for substance nomination.

### Task 2.2: Knowledge management and uptake into policy

Within this task PARC*opedia*, a knowledge management platform, and the long-term strategic roadmap PARC*route* will be developed, with the aim to facilitate the dialogue with regulators. This task aims to deliver PARC*opedia*, a knowledge management platform, that will facilitate the (co-)creation, organisation, contextualisation, and dissemination of the knowledge acquired by the Partnership. It will also integrate knowledge generated in PARC with that from other projects or platforms worldwide, in particular from other Horizon 2020/Horizon Europe projects. PARC*opedia* will allow all parties with an interest in PARC, from within or outside the Partnership and from various domains, to gain optimal access to the content produced by the Partnership.

Additionally, task 2.2 will develop and oversee, as a joint effort with the other WPs, the implementation of a series of strategic roadmaps (PARC*route)* in order to actively promote the uptake of the innovative science and outputs developed in PARC into regulatory risk assessment practice.

### Task 2.3: Sustainability and exit strategy

Task 2.3 will work on the sustainability of a crosscutting and holistic long-term approach for R&I in chemical RA. Countries involved in PARC will establish National Hubs with the relevant ministries, research entities and stakeholders to develop collaboration and contribute to ensuring PARC’s activities are aligned with national activities. Similarly, an EU hub will be set-up. These hubs will also promote the sustainability of the collaboration. Task 2.3 will map their needs, identify the existing expertise, requests and knowledge gaps at national level and feed this into training programmes (WP9) in order to promote the development of the National Hubs.

Through the analysis and discussion of different options for frameworks for long-term sustainability of PARC’s successful activities and the development and monitoring of the PARC performance and impact indicators and impact pathways, task 2.3 will suggest relevant exit strategies that will be the matter of regular discussion at different levels. In close collaboration with different EU DGs and agencies, task 2.3 will also monitor the progress and offer to contribute to international initiatives on chemical risk governance aiming to ensure long-term sustainability and cooperation between different expertise in the field of chemical risk assessment.

## WP3: Synergies, collaborations and awareness

The main goal of WP3 is to boost the impact of PARC through the dissemination of outcomes produced and to foster synergies and collaborations with other initiatives at national and international levels. More specifically, WP3 aims to ensure effective interactions with stakeholders through a Stakeholder Forum and an International Board; to establish a communication and dissemination strategy in order to increase the visibility and impact of PARC among all target groups at the national, European and international level; and to promote synergies and establish effective and efficient collaborations with other relevant scientific and/or regulatory initiatives. PARC’s communication, dissemination and exploitation strategy and plan will be targeted towards the different end-users of the Partnership’s outputs in order to ensure effective and intensive exchanges and engagement to ensure integration of different views.

### Task 3.1: Establishment and running of a Stakeholder Forum and International Board

The Stakeholder Forum will include organisations that represent stakeholders relevant for PARC, such as industry, citizens, patient associations, and trade unions, which are committed to actively interact with PARC. The International Board will consist of experts that have expertise in different areas related to chemical risk assessment, as well as experts in the field of policy development. The International Board, providing a perspective from outside the European Union, will inform on recent developments at the global level and by reflecting on the partnerships activities and results will enhance synergies and innovation. Members of the Stakeholder Forum and International Board will also contribute to the dissemination and uptake of new approaches developed in PARC by regulatory bodies, stakeholder organisations, scientific communities and the public. This will further increase the impact of PARC.

### Task 3.2: Communication, dissemination, and awareness

WP3 is establishing a strategy to ensure timely and effective communication and dissemination of PARC outcomes to research communities and all stakeholders, including the general public, regulators and policy makers. Supported by WP3 with a set of communication tools, the results of the different WPs in PARC will be timely and broadly disseminated to different target groups in order to boost the impact of PARC. The national hubs will play a key role in the communication activities at the national level; thus, their needs will be transposed into the work programme of this WP. In addition, outreach activities are envisaged to allow to uncover citizens’ perceptions and concerns about chemical exposure and to support the development of tailored interventions to raise awareness on chemicals’ risks. These actions will help to build and reinforce the trust that European citizens have in their risk assessment and risk management institutions.

### Task 3.3: Networking and synergies

WP3 is also coordinating the identification of synergies and collaborations of PARC with other programmes and initiatives at the European, national, and regional level. Building on the work undertaken and experience acquired in the HBM4EU, WP3 is developing an overarching framework including a synergies network (gathering information on synergies, collaborations and funding opportunities) complemented with a set of tools to analyse and promote networks, with the aim to avoid too much overlap and to maximise the possibility that PARC outputs are taken up and effectively deployed. Finally, WP3 is establishing a dynamic and coherent communication within the partnership to identify internal needs and gaps, and to promote training and dissemination activities.

## WP4: Monitoring and exposure

**Fig. 2:**
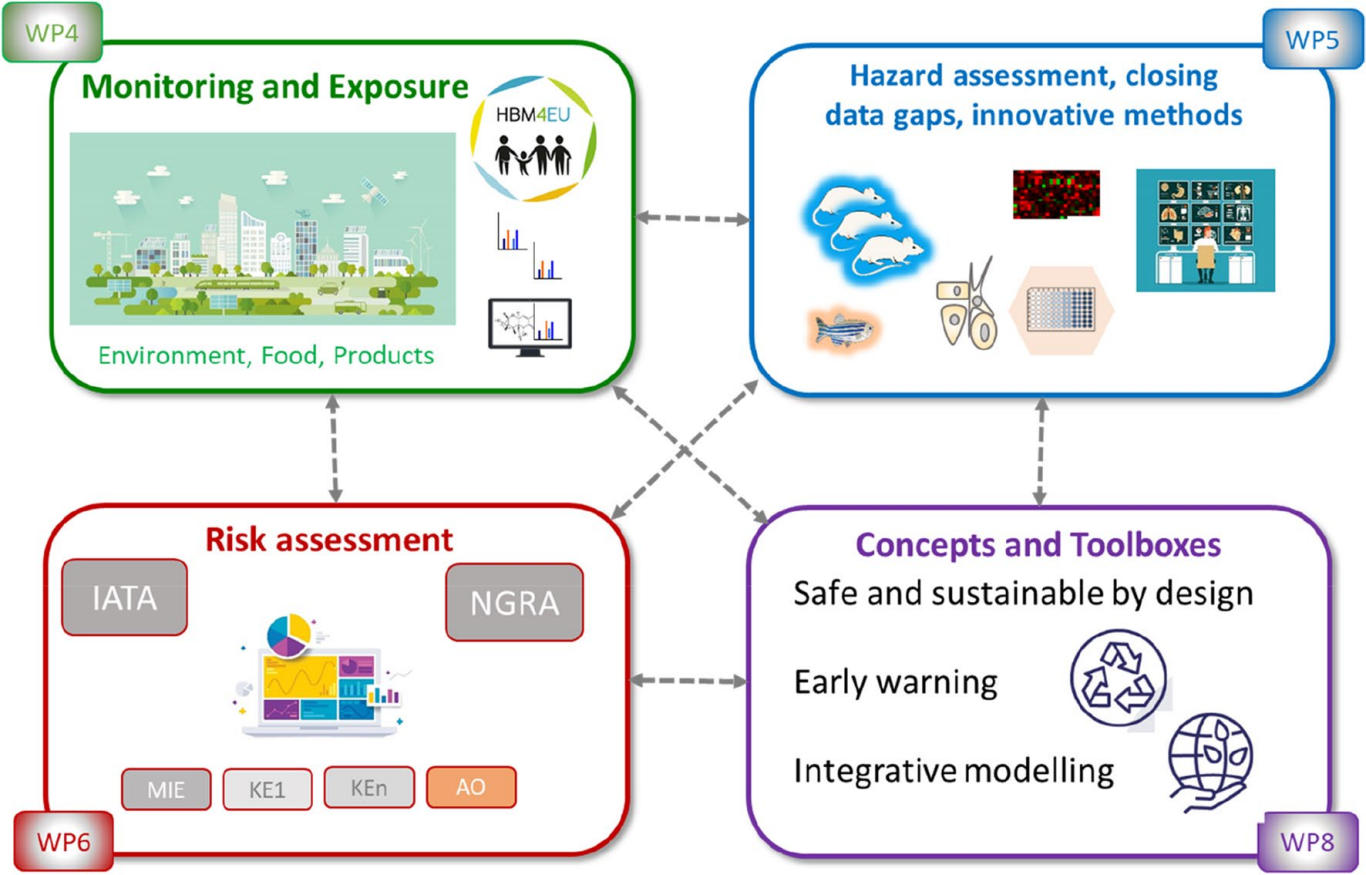
The interplay between the four work packages on exposure (WP4), hazard (WP5) and risk assessments (WP6) as well as safe and sustainable by design (SSBD, WP8)

Guided by regulatory needs, WP4 aims to continue the work of HBM4EU and further develop the chemical monitoring and exposure assessment as well as the respective capacities in Europe as depicted in Fig. 2. The overall goal is a generate reliable Europe-wide FAIR data on human internal and environmental exposure, a better understanding of the environmental and human exposure to chemicals, their interactions, and uptake from multiple sources including pathways of exposure between the environment and humans. In support of a “one substance, one assessment approach” new monitoring approaches will be applied to complement existing monitoring schemes. Robust, reliable and fit-for-purpose innovative tools and methods will be further developed, inter alia to support and facilitate the exposure assessment for vulnerable sub-populations and the early warning detection of chemicals of emerging concern. The work under WP4 is divided into three tasks that address three objectives: (a) continuation of the human biomonitoring platform established by HBM4EU, (b) establishing an EU-wide environmental and multisource monitoring, and (c) further development of innovative methods and tools for monitoring.

### Task 4.1: Human biomonitoring

A HBM survey targeting the general population will form the basis of a well aligned human biomonitoring (HBM) surveillance programme for chemical exposure of European citizens and will generate internal exposure data. Statistically derived internal exposure reference values will allow for the evaluation of spatial and temporal trends in chemical exposure and monitoring of the impact of regulations and the EU’s Chemicals Strategy for Sustainability (EC 2020). In addition, targeted HBM surveys will address specific research and policy questions related to vulnerable groups (young children, pregnant women), highly exposed groups (workers, hotspot residents) or time trends. Priorities will be identified in cooperation with WP2 (‘A common science-policy agenda’). Further to the work achieved in ESBIO (CORDIS), COPHES/DEMOCOPHES (Den Hond et al. 2015; Joas et al. 2012) and HBM4EU (https://www.hbm4eu.eu) and in coordination with WP9 (‘Building infrastructural and human capacities’), activities are being designed and implemented to ensure the quality and comparability of the HBM results. The best suited specific and sensitive exposure biomarkers/matrices and analytical methods for the chemicals to be measured in HBM studies will be identified. The HBM4EU Quality Assurance/Quality Control (QA/QC) programme will be sustained and further developed. New QA/QC approaches to support the use of innovative techniques in HBM surveys will also be explored. Harmonisation and improvement of analytical methods across laboratories will be continued and implemented, defining analytical reference methods when possible. The analysis of exposure and effect biomarkers in the HBM studies will be coordinated and performed by QA/QC qualified laboratories. Special efforts will also be put on the further development of the HBM laboratory network (Esteban Lopez et al. 2021). Task 4.1 will continue the optimisation of the HBM4EU strategy (Apel et al. 2020) to derive Health-Based Guidance Values (HBM-GVs) for the general and/or the occupational population and derive substance specific HBM GV for as many substances investigated in PARC as possible. Systematically identified effect biomarkers will complement exposure biomarkers to support the evaluations of associations between chemical exposure and adverse health effects. Issues related to the linkage of HBM, health examination, occupational and dietary surveys will be identified and solved with support of WP7 (‘FAIR Data’). In addition, innovative tools and methods developed under Task 4.3 will in a first step be evaluated for their reproducibility, relevance and validity and in a second step, if appropriate, be implemented in selected studies to improve sampling strategies and identify emerging chemicals of concern. HBM data will be linked with external exposure data and environmental data generated in Task 4.2 to identify exposure sources, routes, and determinants for internal exposure levels. To identify exposure patterns and associations with health effects, statistical analyses on mixtures will be performed that will feed mixture risk assessment. Task 4.1 will further develop the framework for handling already existing and newly generated data at the national and European level (Gilles et al. 2021), and for data users within PARC. In collaboration with WP2 co-leaders and partners, the ultimate goal of this task is to prepare a long-term sustainable HBM and surveillance system for exposure to chemicals within Europe.

### Task 4.2: Environmental and multisource monitoring

Monitoring studies will be performed to fill gaps in exposure data for an integrated assessment of pollution affecting environmental and human health. In line with the collaborative priority setting, partners will further refine the identified regulatory and research priorities on chemicals, matrices and endpoints following a stepwise approach. The first step will consist of reviewing existing knowledge, activities and infrastructure to build on existing data and identify the availability of samples and state of the art chemical analysis. Based on this review, Task 4.2 will design and conduct monitoring activities with the aim to track the source and route of chemical exposure, supporting the development of an early warning system in WP 8 (‘Concepts and Toolboxes’). This includes defining a sampling strategy, using existing samples and sampling programmes, and selecting analytes and methods. The study design will be developed in collaboration with partners in WP4 to ensure appropriate harmonisation, coordination and/or integration. Particular attention will be paid to a QA/QC concept (in collaboration with the PARC QA/QC Group established in WP9). Data analysis will develop and apply new digitalisation and machine learning-based methods. A Statistical Analysis Group will be set up to ensure harmonised treatment and exploitation of the data. The exploitation of the data could include spatial analysis of exposure levels, input data for exposure factors, evaluation of time trends, comparison with benchmark values for environmental and human health protection, mixture toxicity assessment, integration of chemical data and bioanalytical responses. In collaboration with Task 2.1, a feedback mechanism will be established to analyse whether or not the regulatory needs were met and whether new issues of scientific and/or regulatory significance emerge which should feed into new monitoring activities. Recommendations for integrated monitoring schemes will also be provided.

### Task 4.3: Innovative methods and tools for monitoring and surveys

Innovative (self-)sampling approaches will be evaluated for applicability in large scale monitoring programmes and specific surveys addressing population groups such as the occupational population. Innovative high-throughput in vitro and/or in vivo bioassays for effect-based environmental and human exposure monitoring (Jeddi et al. 2021; Schuijt et al. 2021) will be improved. A framework for Effect-Directed Analysis (EDA), in which suspect screening and non-targeted screening (NTS) based on high-resolution mass spectrometry are embedded to identify the environmental distribution of emerging hazardous chemicals, also as a support to prioritisation in HBM (Pourchet et al. 2020), will be developed. Suspect Screening approaches will be harmonised across the environment-food-HBM communities in terms of definitions, objectives supporting policy context, and technical implementation. NTS approaches will be promoted and harmonised to complement multiple exposure assessment across the environmental, food safety and HBM fields through new exposure markers. For higher efficiency and deployment, advanced data processing methodologies and bioinformatic tools will be established and the quantitative performances of NTS to comply with regulatory needs and the throughput, necessary to allow implementation in larger studies, will be improved. Building on work performed in HBM4EU, common QA/QC requirements will be defined for effect-based/EDA/Suspect Screening/NTS approaches applied to environmental, food or human matrices, and corresponding technical guidelines will be elaborated. These innovative approaches will be evaluated through complementary proof-of-concepts each dealing with a given particular new sampling and measurement approach applied to a given exposure/sample type and sub-population.

## WP5: Hazard assessment

The overall goal of WP5 is to overcome the major challenges in hazard assessment for human and environmental health as depicted in Fig. 2. The specific objectives are: (a) to close data gaps identified by key stakeholders; (b) to improve the current hazard characterisation paradigm by establishing comprehensive testing strategies, thereby promoting the availability and applicability of new approach methodologies (NAMs) in risk assessment; (c) to contribute to the improvement of mechanistic understanding of toxicity by analysing all available data and applying systems toxicology approaches and taking into account AOPs and to improve modelling approaches such as PBPK modelling. The work envisaged is divided into three tasks.

### Task 5.1: Closing data gaps of concern

This task aims to investigate and close existing data gaps through toxicity testing. Following a prioritisation exercise and further consultation with partners involved in hazard and risk assessment in national and European agencies, the following groups of substances have been selected for the first round of testing: natural toxins, in particular the mycotoxins enniatins and those derived from *Alternaria*, as well as alternatives to bisphenol A (BPA). In contrast to man-made chemicals, toxins do not have a producer that has to provide data as part of a legal approval procedure, but their presence in food or consumer products is of concern. Consequently, regulatory bodies recognised a strong need for data in this area. Activities on toxins such as mycotoxins of the *Alternaria* or enniatins group will address existing data gaps left open by regulatory data requirements, such as toxicokinetics data or comprehensive studies like the Extended One-Generation Reproductive Toxicity study (OECD 2018a). The latter study is also envisaged for other groups of substances of concern, which may include (but are not limited to) synthetic contaminants such as BPA alternatives/analogues not assessed by industry for authorisation or PFAS (per- and polyfluoroalkyl substances).

### Task 5.2: Innovative tools and methods for toxicity testing

The focus of this task is on the improvement of the current hazard characterisation paradigm by establishing comprehensive testing strategies that logically combine novel methods with well-established approaches, preferably in a tiered manner. The regulatory needs to address specific toxicological endpoints are anchored in different regulations including the Classification, Labelling and Packaging (CLP) Regulation (EC 2008), cosmetics Regulation (EC 2009a; which relies on NAMs only), the plant protection products (EC 2009b) and biocides regulations (EU 2012; aiming at mechanistic data e.g. in the field of endocrine disruption), food contact materials (EC 2004; aiming at identifying effects of non-intentionally added substances) or the REACH Regulation (EC 2006; aiming at closure of multiple data gaps by applying data based read-across and grouping approaches) as well as the EU Chemicals Strategy for Sustainability (EC 2020).

This task will address (obvious) gaps in knowledge from multiple perspectives. In brief, this task will evaluate the relevance and readiness of new technologies, e.g. genomic, transcriptomic, proteomic, high-content analysis microscopy, high resolution mass spectrometry, and human inducible pluripotent stem cell technology, for the assessment of (eco)toxicological endpoints such as non-genotoxic carcinogenicity, immunotoxicity, endocrine disruption and (developmental) neurotoxicity, thereby leveraging OECD’s and PARC’s AOP frameworks. Also, test methods, tools and methodologies will be developed to identify drivers of toxicity in mixtures and to support the grouping of chemicals, including the application of read-across. Furthermore, this task aims for the development and application of predictive in vitro and in silico tools to identify and characterise specific hazards. Through close collaboration with human biomonitoring communities in- and outside PARC, methods and computational approaches will be developed to study the metabolism of chemical substances in different biological (test) systems or matrices (e.g. cells, organoids, organs, blood) for comparison between species.

### Task 5.3: Quantitative systems toxicology and AOP development

Enhancing the understanding of mechanisms underlying toxicity will be achieved by analysing available data and applying systems toxicology approaches. This task will contribute to the development of AOPs and provide data for the improvement of Physiologically Based Toxicokinetic (PBK) models. It further aims to integrate relevant human disease models and develop concepts facilitating in vitro—in vivo extrapolation (IVIVE). For this, systems biology methodology will be applied to in silico*, *in vivo and in vitro data (biochemical data, omics, endpoints). New data generated in tasks 5.1 and 5.2 as well as relevant data from databases will be mapped to existing (networks of) AOPs, with the aim to hypothesise adverse outcomes. Available literature will be automatically explored to fill gaps in AOPs and to link priority chemicals to events in AOPs using text mining and network analysis tools. This will support the identification and biological characterisation of effect markers that can, later on, be used in WP4. Human omics and other relevant datasets on human pathophysiology of relevant disease states from public domain sources will be integrated. Using bioinformatic tools, this will allow to inform human disease mechanisms based on omics and other information. Gaps in pathophysiological knowledge will then be filled by using datasets from existing human biobanks. Finally, WP5 will correlate in vitro and in vivo mode of action (MoA) with human data.

Regarding PBK models and quantitative systems toxicology, Task 5.3 aims to develop IVIVE-PBK models, to characterise the impact and predictivity of in vitro Absorption, Distribution, Metabolism and Excretion (ADME) parameters, to quantify the uncertainty by comparing to in vivo ADME studies and to collaborate with WP6 on a wide range of case studies. Toxicokinetic parameters will be generated for all WP5 test compounds using the appropriate in vitro test systems, in silico predictions and analytical measurements. PBK modelling will be used to estimate internal exposure and to compare this to Points of Departure derived from in vitro mechanistic NAM assays for priority chemicals and pathways. Integration of toxicokinetic and toxicodynamic models in quantitative systems toxicology models will improve adverse outcome prediction.

## WP6: Innovation in regulatory risk assessment

The overall goal of WP6 is to drive innovation in regulatory risk assessment by strengthening its scientific basis, with implementation of NGRA as ultimate goal. WP6 will consider both improvements that are achievable in the near future and improvements that would require additional knowledge and policy development for implementation. The aim of WP6 is to develop and implement the best scientific achievements in the risk assessment processes as well as responding to the needs of the regulatory and policy community to drive innovation in the regulatory risk assessment process. By developing chemical risk assessment science through a system-thinking approach, combining and connecting the best of different scientific domains, and sharing common visions and roadmaps, WP6 will contribute to a transformational change in the field of chemical risk assessment and support the ‘one substance, one assessment’ approach. The work in WP6 is divided into four tasks.

### Task 6.1: Integrated approaches to testing and assessment of chemicals

The goal of this task is to establish IATAs (Integrated Approaches to Testing and Assessment) (OECD 2020), to be used for different European regulations on chemical safety for different industry sectors as well as for the CLP Regulation (EC 2008). The IATAs will be developed for a selected set of health effects and evaluated through dedicated case studies. To provide a strong mechanistic basis for IATA development, existing and newly developed AOPs for selected health effects will be combined into AOP networks. Wherever possible and relevant, Key Event Relationships will be quantitated by applying computational methodologies. IATAs will be developed in close collaboration with stakeholders including OECD and through various cycles of optimisation; they may include tiered approaches, ranging from high-throughput approaches to complex in vitro models. In addition, systematic quantitative determination of the uncertainty of application of a combined set of NAMs in an IATA will provide insight in the overall confidence for the application of a selected test battery for respective IATAs. The outcome of these activities will feed back into IATA development as part of an iterative process of IATA improvement.

### Task 6.2: Integrative exposure and risk assessment

The aim of this task is to develop innovative and practical approaches for human health risk and impact assessment of single, aggregated and combined exposure to chemicals via multiple sources across regulatory silos and routes during lifetime, and to foster their regulatory uptake. Relative contributions of sources and routes will be determined from aggregate exposure to support effective RM measures. Living and working environment as well as chemical transfer from soil to water, food, and air and migration from consumer products, articles and materials will be integrated to advance knowledge on the exposure sources. For estimating and reconstructing chemical exposure through life, accounting for varying exposure scenarios and physiological and biochemical characteristics over time, PBK models will be developed to address the different sensitivity of humans within a population. Simulations of concentrations in target tissues will be linked to AOPs and IATAs. Finally, data availability and methodologies to perform health impact and cost–benefit assessment for prioritised chemicals, exposure routes, windows of exposure, subpopulations, geographical locations, and health outcomes will be improved. A selection of indicators integrating dynamic exposure and hazard data and accounting for variability and uncertainties will be developed to estimate the health risk and impact of specific exposure scenarios.

### Task 6.3: Review of risk assessment methodology

Review of current regulatory risk assessment methodologies within and across relevant chemical sectors will be performed through a series of case studies. Effectiveness of the methodologies will be reviewed. The work performed will contribute towards a science-based, coherent, and transparent assessment of chemicals, considering available tools, criteria, and methods, to reduce the underlying uncertainty and better protect human health, including workers, and the environment. The case studies will provide science-based support for method development, identify R&I needs as well as suggest improvements and harmonisation opportunities. For the first years, the case studies will focus on two areas: substance and effect specific reviews and use of tools, criteria, and methods. Thereafter the focus areas will be evaluated, and either be further analysed through additional case studies or, if relevant, replaced with new focus areas. Final conclusions and recommendations based on the case studies performed will be generated, also considering the EU-wide relevance.

### Task 6.4: Transposing results to regulatory risk assessment methodologies

This task will support regulatory processes for reducing risks to humans and the environment by undertaking scenario-based case-studies resulting in guidance documents and/or recommendations. First, in coordination with ongoing EC activities, an European framework for the regulatory assessment of mixtures encompassing all relevant chemical classes, exposure scenarios and protection goals will be developed. It will build a consistent overarching scientific framework that can be adapted to the different protection goals, different chemicals, data situations and regulatory contexts. It will evaluate the availability and quality of data for regulatory assessment, analyse the additional risk caused by mixtures and its implications. The second activity aims to promote and facilitate the regulatory acceptance and practical use of NAMs in risk assessment across different chemical sectors. Focus will be put on real-life situations and application contexts encountered by risk assessors. Long-term objectives include the definition of harmonised scientific criteria for acceptance of NAMs across regulations, and of an evidence-based framework to ensure systematic, transparent, and reproducible application of NAMs in risk assessment. Next, we will develop and apply tools and databases that enable a systematic identification of priority substances in products and materials. In doing so, the activity will promote a more effective and harmonised enforcement of legislation, support informed substitution of problematic chemicals in products and materials and inform how sustainability assessments can be achieved with respect to chemicals. Finally, we will explore in close cooperation with regulatory risk assessors risk assessment methods to improve Environmental Risk Assessment of plant protection products and to overcome shortcomings in the current substance-by-substance paradigm.

## WP7: FAIR data

The aim of PARC is to enable all the scientific communities involved in chemical risk assessment and risk management, as well as stakeholders, to have access to high-quality data by facilitating their accessibility, interoperability and usefulness for research. In the context of the EU’s Open Science Policy (European Union 2020a), PARC will promote harmonisation of data and exchange between different actors (scientific community, health agencies, regulators, policy-makers etc.) and disciplines (exposure science, toxicology…) to promote transparency, support risk assessment, and allow for reuse. It will ensure data and associated information is FAIR, i.e. Findable, Accessible, Interoperable, and Reusable, and addresses the General Data Protection Regulation (GDPR; EU 2016) related challenges for data exchange.

### Task 7.1: PARC FAIR Data Policy (PFDP) and Data Management Plan (DMP)

Data, in particular accessibility and interoperability, is a major challenge. Data “FAIRification” will be a key activity of PARC to facilitate data reuse. Implementing FAIR data practices is among the main operational objectives of PARC and will be measured by the proportion of datasets developed in PARC that will be FAIR, and the number of deliverables reusing existing data. A common Data Management Plan (DMP) will be established and shared.

The PARC FAIR data policy (PFDP) will define the principles and conditions that govern provision, management, access, use and re-use of data, in line with the FAIR principles, while taking into account legal aspects (i.e. GPDR), security, transparency, sustainability and quality of the data. WP7 will provide the framework (guiding principles, data policy, framework DMP), set up specific methods and tools and guidance to FAIRify data and facilitate data reuse in the R&I WPs and in regulatory agencies. Through training, FAIR implementation will be broadly embedded in PARC. PARC will establish collaboration with specialised FAIR initiatives and projects (e.g. EOSC-Life, ENVRIFAIR, WorldFAIR), and collaborate with experts from the Go FAIR Foundation to kick start the FAIRification process in the first two years.

### Task 7.2: Data libraries

Data will be made FAIR at the source whenever possible, described by FAIR metadata, and associated with a persistent identifier. PARC will use established FAIRification approaches such as the Three Point FAIRification framework (3PFF), elaboration of FAIR Implementation Profiles (FIPs) defining concrete implementation choices for each of the 15 FAIR principles (Schultes et al. 2020; Wilkinson et al. 2016), and established software solutions to manage FAIR data sources. Specific use cases will enable the multi-faceted FAIRification process to be developed gradually and pragmatically. Compatibility assessment, efforts for harmonisation and maximum interoperability will start from these FIPs. Standardised templates will be created, as necessary, based on domain specific standards, formats and vocabularies whenever available. PARC will perform landscape exercises and FAIRness assessments to identify potential partners to functionally integrate and exchange different types of data from different domains, and different national and transnational levels. It will build further upon DG ENV’s Common Data Platform for Chemicals (CPCD), which will be the default option for data that are currently used in standard RA. A joint exercise will be set up to align PARC activities with the CPCD roadmap. For data that are currently not commonly used in regulatory risk assessment, the content and accessibility of specialised data centres (e.g. ESFRI (European Strategic Forum for Research Infrastructure) research infrastructures (i.e., EIRENE, ELIXIR), MS Open Data initiatives, domain-specific repositories, institutional repositories, open generic repositories such as Zenodo) will be explored. Furthermore efforts and achievements from recently ended projects such as EuroMix (https://www.euromixproject.eu/) and HBM4EU (https://www.hbm4eu.eu/), ongoing project clusters such as the European Human Exposome Network (EHEN), cluster or the European Cluster to Improve Identification of Endocrine Disruptors (EURION), and community databases such as NORMAN (https://www.norman-network.net/) will be integrated. The ECs Information platform for Chemical Monitoring (IPCHEM) and the OECD eChemPortal will be implemented through semantic mapping of the underpinning data schema. A dedicated FAIR data hub hosting core functionalities (such as querying of data, use of searchable catalogues and metadata services) complementary to existing platforms, will be implemented. Establishment of new FAIR Data Points (FDPs) (Thompson et al. 2020) or dedicated repositories will be considered in cases where existing solutions do not satisfy the needs of the PFDP.

### Task 7.3: Innovative analyses

Innovative analytical approaches to maximise use and insights from increasing amounts of open and FAIR data for RA will be introduced. Promising approaches will be applied to use cases, selected together with WPs 4–8, to evaluate and showcase their usefulness. A range of advanced approaches will be developed and documented, including pooling and meta-analysis of heterogeneous datasets, uncertainty analysis, and knowledge and text mining via on-the-fly ontological mapping, Bayesian networks, machine learning and other approaches. Analytical tools will be made available as FAIR algorithms and supplied to WPs 4–8 for use and integration into toolboxes. Good practice guidelines describing the approaches and their applications for regulatory risk assessment will be published.

## WP8: Concepts and toolboxes

The overall goal of the WP8 in PARC is to support the development and consolidation of new concepts and tools that address key challenges of the Chemicals Strategy for Sustainability (EC 2020). These include (a) supporting the operationalisation of the framework for Safe and Sustainable by Design (SSbD) chemicals and materials; (b) contribute to the EC’s Early Warning System (EWS) for chemicals of emerging concern; and (c) risk modelling integration into a network of state-of-the-art computational tools. The work is articulated in the following three tasks.

### Task 8.1: Safe and Sustainable by Design (SSbD)

The aim of this task is to support the operationalisation of the SSbD criteria and methodology developed by the EC by gathering feedback from various stakeholders, including industry, on its operational applicability, and by developing and testing a toolbox geared to support the implementation of SSbD by the various users. For this, PARC will function as a sounding board for the EC's ideas on SSbD by collecting reflections of scientists and other users on the applicability of SSbD for chemical risk assessment, and vice versa, and putting it in a practical perspective to define the requirements for a toolbox. Use cases in various sectors will be selected to test the SSbD practical applicability through a learning-by-doing approach. Towards this goal a knowledge and information platform will be developed and established as part of PARC*opedia* dedicated to SSbD, and in connection to existing knowledge sharing initiatives. Educational material will be produced to ensure that innovators and younger generations will embed SSbD in their mindset and way of working. Indicators to measure progress on sector applicability of the SSbD toolbox will be developed to support the EC monitoring activities towards SSbD implementation.

### Task 8.2: Scientific and technical basis for an early warning system (EWS) on chemical risks

The major aim of this task is to feed into the EC’s early warning and action system, announced under the Chemicals Strategy for Sustainability. This task involves development and validation of (a) early warning monitoring tools in humans, environment and products combining exposure and hazard data, (b) effect-directed tools based on bioassays using new approach methodologies (NAMs) taking stock of the relevant JRC work, (c) suspect and non-target screening tools, (d) effect-directed analysis (EDA), (e) metabolomics-based biomarkers, and (f) machine learning for pattern recognition of potential vectors that would need further scrutiny from big and complex data sources, including system modelling. A close link with ECHA and its relevant databases will be established.

An identification framework will be developed to identify new hazardous substances, their sources and transformation products. Therefore, a wide range of methodologies will be applied to prioritise new toxic drivers, delivered through toxicological assessment in PARC, including NAMs. The early warning methodology and toolbox will be tested for applicability and performance through case studies to develop an operational system supporting regulatory frameworks. The substances from the early warning tools and framework will be ranked based on their potential risks and integrative models and be made publicly available for dissemination to policy-makers, regulators and other stakeholders.

### Task 8.3: Integrative models

This task aims to place the models developed and the data that will be collected and generated within PARC in an overarching and harmonised framework and to implement this in a functional and open PARC computational tool network infrastructure, including links to the SSbD toolbox and models from other projects. PARC modelers will be organised in a network to produce an inventory of models and discuss technical integration of models and tools. Major stakeholders, such as EFSA, ECHA and relevant national agencies will be involved. Furthermore, the role of uncertainty and data harmonisation aspects will be considered in order to assess different methodological frameworks at each step of the risk assessment process, as well as to quantitatively estimate and reduce uncertainties relevant for different risk assessment approaches. Particular attention will also be paid to compliance with FAIR principles and establishing a harmonised way of communication between the nodes of the PARC model network. This will enhance the generic character of the PARC developed integrative model network infrastructure, for a broad range of risk assessment requests, including the use cases defined.

## WP9: Building infrastructural and human capacities

The aim of WP9 is to facilitate the establishment and maintenance of the capacities needed to address current, emerging, and novel challenges in chemical hazard, exposure, and risk assessment to support NGRA strategies and ultimately reach the zero-pollution ambition for a toxic-free environment. This will be achieved by (a) inventorying existing monitoring and biomonitoring networks, environmental specimen banks, laboratory and sampling networks and capacities and other resources in various chemical risk assessment domains, (b) identifying gaps and designing activities contributing to filling such gaps, (c) developing and coordinating the joint activities to strengthen capacities, and (d) setting up a training network for the PARC consortium and the risk assessment and risk management communities. The work envisaged for WP9 will be addressed in the following tasks.

### Task 9.1: Laboratory networking

Built on the work done and experience from HBM4EU (https://www.hbm4eu.eu/) regarding development of laboratory networks in human biomonitoring and based on existing well-established laboratory networks, task 9.1 aims to expand laboratory capacities. The existing networks in specific domains, such as human biomonitoring, environmental monitoring, food and feed, both target and non-target analysis, toxicology and ecotoxicology, in vivo and in vitro methods will be mapped. Following a dedicated analysis of possible gaps, strategies for the establishment and/or strengthening of laboratory networks, through coordination activities, will be defined.

### Task 9.2: Building exposure monitoring capacities

Task 9.2 will map environmental (air, water, sediment, soil, biota), drinking water, food and feed monitoring networks in both the regulatory and research areas, biomonitoring efforts and human cohorts, and identify the gaps and opportunities for future development and/or alignment. The task will build on collaboration with WP3 stakeholder surveys and align with data requirements set up in WP7. Both task 9.1 and 9.2 will capitalise on the existence of European and national laboratory and data networks and international programmes, projects, clusters and infrastructures as well as other scientific networks building on their experience and knowledge.

### Task 9.3: Joint activities – harmonisation

Task 9.3 aims to harmonise laboratory related Quality Assurance /Quality Control across application, technique and chemical domains by implementing joint activities. Currently the state of maturity varies greatly between food (well established EU/National Reference Laboratory networks through legislation) and HBM (only recently being established in HBM4EU), as well as toxicology laboratories. Across and even still within the more mature domains, definitions, interpretations, and procedures for e.g. performance parameters and assessment of interlaboratory comparability differ, and are not necessarily consistent. With chemical risk assessment developing towards a “one health” approach, it is essential that generation of all laboratory data is based on the same principles, and according to the same procedures as much as possible. This task will build upon the existing data harmonisation and quality assurance/control tools such as the DG JRC Certified Reference Materials.

### Task 9.4: Training

Task 9.4 aims at setting up a training network for both PARC members, risk assessors and managers. Training needs will be identified in close collaboration with WP2 (national hubs), WP3 (stakeholders´ surveys), and WPs 4, 5, 6, 7 and 8 (internal training). The training programme will benefit from existing training networks and fellowship programmes for identified topics (e.g., risk assessment; data management; risk communication; modelling) as well as tailor-made PARC training modules, making guidance on training programmes, opportunities and training materials available on a web-based portal.

## Discussion and conclusion

Exposure monitoring, hazard and ultimately risk assessment of chemical substances or combinations thereof is in high need of a paradigm shift. Over the past years, a number of (research) projects and initiatives have made progress in this field. This includes, but is not limited to, US American initiatives like ToxCast, the US National Toxicology Program (NTP), the Endocrine Disruptor Screening program (EDSP), international initiatives at the level of the WHO International Program on Chemical Safety (IPCS) and the OECD. On top of that, various EU-funded projects have been conducted, such as the SEURAT initiatives (Daston et al. 2015; Gocht et al. 2015), EUToxRISK (Escher et al. 2019; Krebs et al. 2020; Mone et al. 2020), HBM4EU (Ganzleben et al. 2017; Kolossa-Gehring et al. 2023), EuroMix (Rotter et al. 2018) and many more. They all had success with respect to their aims and goals; nevertheless, the actual implementation of NGRA into regulatory practice is still lagging. This is due to several reasons, with one of them being that the regulatory community has not been fully involved in most of these projects or initiatives. PARC, given its core involvement of regulatory agencies at the EU and national level as well as its unprecedented scale, has the potential to make a difference in this regard, at least for Europe. Through the establishment of an EU-wide research and innovation hub of excellence to support EU and national chemical risk assessment and risk management authorities to address current, emerging and novel chemical safety challenges, PARC will enable the transition to NGRA. The production of data and knowledge will enable RA institutions to give better advice to risk managers and decision-makers. PARC’s success depends on whether its outcomes are accepted and exploited by end-users, notably policy-makers, to improve chemical risk assessment and management. To raise awareness and ensure that outcomes are useful and taken-up, end-users, and in particular policy-makers, are involved in all stages through an iterative consultation process.

This is further underpinned by the fact that all elements relevant for chemical risk assessment, i.e. hazard identification, hazard characterisation, exposure assessment and risk assessment, are incorporated in the blueprint of PARC and a wide range of skills and expertise is represented in the consortium and in particular in Management Board through the extensive and complementary expertise of the WP leaders. This ensures not only a smooth exchange of information and close collaboration between all partners involved, who share the common goal to move towards NGRA, but also ensures the coherence and integration of the different activities and synergies implemented. An expected co-benefit of a durable programme for chemical risk assessment coupled to the science-to-policy and capacity-building activities implemented in PARC shall also reinvigorate the human and environmental exposure, toxicology and ecotoxicology research community in Europe and contribute to developing European capacities in these domains while ensuring cross-disciplinary and cross-cutting discussion and understanding.

At the same time, the complexity of the challenge ahead is well understood and the PARC consortium is fully aware that not all issues related to putting NGRA into practice will be resolved by this partnership. Nevertheless, we are confident that through close collaborations with related initiatives and projects around the globe and the interaction with stakeholders representing all interesting parties from industry to citizens PARC will be able to make a significant contribution. On the longer term, PARC will yield a community across Europe, including next-generation professionals, that has ample expertise in chemical risk assessment and interest in an efficient science to policy dialogue and interface required to apply and contribute to the long-term visions of European policies such as the Green Deal and the Chemicals Strategy for Sustainability and their cross-linked actions. This will further improve regulatory decisions at the European level and facilitate the operationalisation of the “one substance, one assessment” concept.
